# Diffusion along the perivascular space influenced by handedness and language lateralisation

**DOI:** 10.1093/braincomms/fcaf252

**Published:** 2025-06-24

**Authors:** Gábor Perlaki, Barnabás Dudás, Réka Horváth, Gergely Orsi, Gergely Darnai, Ákos Arató, Szilvia Anett Nagy, Tamás Dóczi, Sámuel Komoly, Norbert Kovács, József Janszky

**Affiliations:** HUN-REN-PTE Clinical Neuroscience MR Research Group, H-7623 Pécs, Hungary; Department of Neurology, Medical School, University of Pécs, H-7623 Pécs, Hungary; Department of Neurosurgery, Medical School, University of Pécs, H-7623 Pécs, Hungary; Pécs Diagnostic Centre, NeuroCT Ltd., H-7623 Pécs, Hungary; Department of Neurology, Medical School, University of Pécs, H-7623 Pécs, Hungary; Department of Neurology, Medical School, University of Pécs, H-7623 Pécs, Hungary; HUN-REN-PTE Clinical Neuroscience MR Research Group, H-7623 Pécs, Hungary; Department of Neurology, Medical School, University of Pécs, H-7623 Pécs, Hungary; Department of Neurosurgery, Medical School, University of Pécs, H-7623 Pécs, Hungary; Pécs Diagnostic Centre, NeuroCT Ltd., H-7623 Pécs, Hungary; Department of Neurology, Medical School, University of Pécs, H-7623 Pécs, Hungary; Department of Behavioural Sciences, Medical School, University of Pécs, H-7624 Pécs, Hungary; Department of Neurology, Medical School, University of Pécs, H-7623 Pécs, Hungary; HUN-REN-PTE Clinical Neuroscience MR Research Group, H-7623 Pécs, Hungary; Department of Neurology, Medical School, University of Pécs, H-7623 Pécs, Hungary; Pécs Diagnostic Centre, NeuroCT Ltd., H-7623 Pécs, Hungary; Structural Neurobiology Research Group, Szentágothai Research Centre, University of Pécs, H-7624 Pécs, Hungary; Department of Neurosurgery, Medical School, University of Pécs, H-7623 Pécs, Hungary; Pécs Diagnostic Centre, NeuroCT Ltd., H-7623 Pécs, Hungary; Department of Neurology, Medical School, University of Pécs, H-7623 Pécs, Hungary; Department of Neurology, Medical School, University of Pécs, H-7623 Pécs, Hungary; HUN-REN-PTE Clinical Neuroscience MR Research Group, H-7623 Pécs, Hungary; Department of Neurology, Medical School, University of Pécs, H-7623 Pécs, Hungary

**Keywords:** DTI-ALPS, diffusion, glymphatic system, language lateralisation, handedness

## Abstract

The DTI-ALPS_index_ (diffusion tensor image analysis along the perivascular space index) has been tested in several diseases, but a limited number of studies are available in healthy normal populations. Given that some of the examined diseases show asymmetric features (e.g. Parkinson’s disease, epilepsy) and considering that the DTI-ALPS_index_ is calculated from white matter regions involved in motor and language functions, our aim was to examine whether the index is influenced by handedness and/or functional language lateralisation. Eighty-two Caucasian adults (35 males; mean age: 26.91 ± 10.17, range: 18–66 years) were included in this retrospective cross-sectional study. Handedness was assessed by the Edinburgh Handedness Inventory, while language lateralisation was determined by functional MRI at 3T using a verbal fluency paradigm. The left- and right-hemispheric as well as the bilateral DTI-ALPS indices were calculated independently by two observers. Multiple linear regression corrected for age and sex indicated that handedness was inversely related to the left ALPS_index_ by Observer1 (*n* = 81, two-tailed *P* = 0.030) and the bilateral ALPS_index_ by both observers (*n* = 82, *P* = 0.025 and *P* = 0.044), but not significantly related to the right ALPS_index_. In females, multiple linear regression corrected for age indicated that language laterality index was inversely related to the left ALPS_index_ (*n* = 47, *P* < 0.001) and the bilateral ALPS_index_ (*n* = 47, *P* ≤ 0.003) by both observers, but not significantly associated with the right ALPS_index_. In males, language laterality index was not related to any of the ALPS indices. Providing further insights into the normal DTI-ALPS patterns, our study supports the hypothesis that both language laterality and handedness can affect DTI-ALPS index that should be considered in future studies.

## Teaser

Perlaki *et al*. report that both handedness and language lateralisation influence the MRI-based index of diffusion tensor image analysis along the perivascular space, that should be considered by future studies. The findings offer new insight into normal patterns of the index in healthy population.

## Graphical Abstract

**Graphical Abstract:**
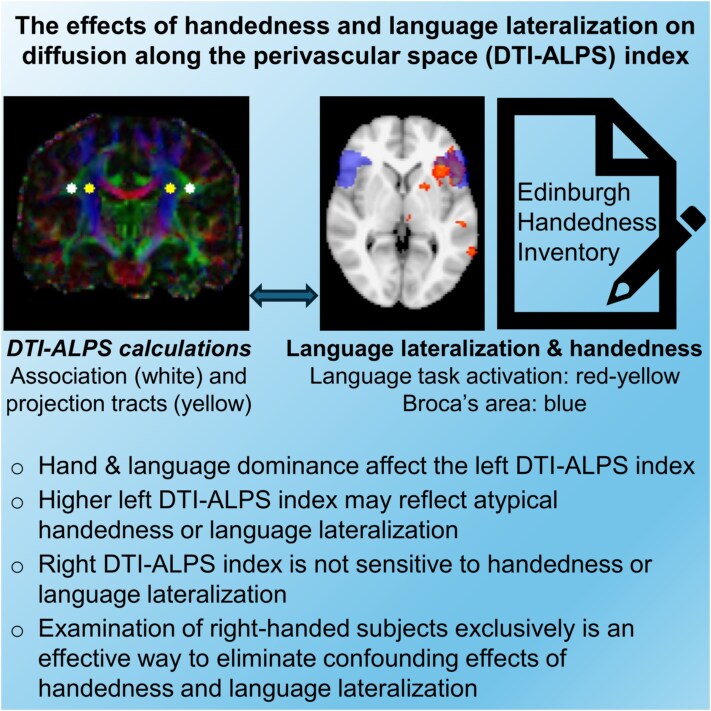

## Introduction

Although it was historically thought that there is no lymphatic system in the brain,^1^ animal studies demonstrated an aquaporin 4-mediated paravascular pathway, contributing to the clearance of interstitial solutes/wastes from the brain (referred to as glymphatic system),^2^ in addition to confirming the existence of meningeal lymphatic vessels.^3^ As demonstrated by both small animal and human studies, the newly proposed ‘glymphatic system’ can potentially be assessed using intrathecal contrast-enhanced MRI.^4,5^ However, due to the associated health risks and time-consuming process associated with this invasive method, it has not become widespread to study the human glymphatic system.^6,7^

The method of DTI-ALPS (diffusion tensor image analysis along the perivascular space) index was introduced by Taoka *et al*.^8^ in 2017 with the intention of enabling a more extensive non-invasive study of the glymphatic system. Importantly, in this initial study, the index was only theoretically speculated to be associated with glymphatic function, and although later Zhang *et al*.^9^ showed its correlation with invasive contrast-enhanced glymphatic MRI measurements, at the current state of our knowledge, its rigorous validation as a marker for glymphatic system is still missing.^10^ Some recent studies suggested that changes in the ratio of radial diffusivities of the diffusion tensor (i.e. λ_2_/λ_3_) occurring with aging and neurodegeneration, as well as the presence of crossing fibres may bias the ALPS index.^11,12^ While the index is often handled as a synonym for glymphatic function and interpreted accordingly, given criticisms of this interpretation and ongoing research on its exact underlying/confounding factors, without further validation, any claims linking the ALPS index to glymphatic function should be approached with caution.^13^ Nevertheless, the ‘DTI-ALPS index’ is fortunately become a widely established well-known term that could be interpreted in its own right (i.e. increased/decreased ALPS index), as suggested.^13^

The favourable reproducibility, intra- and inter-rater reliability of DTI-ALPS index have been shown, and the non-invasive method has been used in a number of clinical conditions, such as Alzheimer’s disease,^8^ multiple sclerosis,^14^ mild traumatic brain injury,^15^ epilepsy^16,17^ or Parkinson’s disease.^18^ Even though DTI-ALPS has been studied for many other disease-related conditions, a limited number of studies are available in healthy normal population. Many studies observed an inverse association between age and DTI-ALPS index, but the influence of sex is rather still under investigation.^19,20^ Furthermore, as far as we know the potential influence of handedness or language lateralisation on the DTI-ALPS index has not yet been studied and without understanding their potential confounding effects, some of the studies calculated an index averaged across both hemispheres,^9,21^ while others obtained measurements only from the left hemisphere ‘justified’ by the right-handedness of their subjects.^8^

Considering that some of the above-mentioned diseases show asymmetric characteristics (e.g. Parkinson’s disease, epilepsy), and the fact that DTI-ALPS index is calculated from white matter regions involved in motor and language functions (i.e. corona radiata and superior longitudinal fasciculus),^21^ examining the influence of handedness and language dominance on DTI-ALPS index may be an essential step to extend our understanding of normal DTI-ALPS patterns in healthy populations. Moreover, investigating the normal variations of DTI-ALPS index is extremely important to avoid misinterpretation of the results found in patient groups. The aim of the present study was to examine whether DTI-ALPS index is influenced by handedness or language lateralisation.

## Materials and methods

### Subjects

Data for this retrospective study were collected from individuals without known neuropsychiatric diseases or MRI abnormalities, who underwent both DTI (diffusion-tensor imaging) and task-based fMRI (functional MRI) for language lateralisation assessment according to the same imaging protocol between 2009 and 2013 at the University of Pécs as part of a larger study focusing on handedness and language dominance. Of the 86 subjects in this group, four were excluded due to insufficient fMRI data (i.e. missing activations in the Broca’s area in both hemispheres or serious movement artefacts). The final sample included 82 Caucasian adults (35 males; mean age: 26.91 ± 10.17, range: 18–66 years). The handedness of all subjects was assessed by the Edinburgh Handedness Inventory (EHI).^22^ All participants provided written informed consent in accordance with the Declaration of Helsinki. The retrospective study was approved by Regional Ethical Board of the University of Pécs (9910-PTE 2024).

### Imaging data acquisition

All imaging data were acquired on the same Siemens TIM Trio 3T MRI scanner with a 12-channel head coil. Functional MRI data were measured using a 2D gradient-echo EPI sequence (TR/TE = 2000/36 ms; Flip Angle = 76°; 23 axial slices; slice thickness = 4 mm; no interslice gap; FOV = 192 × 192 mm^2^; matrix size = 92 × 92; voxel size = 2.1 × 2.1 × 4 mm^3^; receiver bandwidth = 1360 Hz/pixel; interleaved slice order to avoid crosstalk between contiguous slices; 210 volumes). A 2D diffusion-weighted spin-echo EPI sequence was used for diffusion tensor imaging measurements (TR/TE = 6700/78 ms; 60 axial slices; slice thickness = 2 mm; no interslice gap; FOV = 211 × 260 mm^2^; matrix size = 104 × 128; voxel size = 2 × 2 × 2 mm^3^; diffusion gradients were applied in 20 directions with a *b*-value of 700 s/mm^2^ and a single volume was collected with no diffusion gradients applied; bandwidth = 1698 Hz/pixel; number of averages = 3).

A 3D T1-weighted axial (TR/TI/TE = 1900/900/3.41 ms; Flip Angle = 9°; 144 axial slices; slice thickness = 0.9 mm; no interslice gap; FOV = 201 × 230 mm^2^; matrix size = 224 × 256; voxel size = 0.9 × 0.9 × 0.9 mm^3^; receiver bandwidth = 180 Hz/pixel) or sagittal (TR/TI/TE = 2530/1100/3.37 ms; Flip Angle = 7°; 176 sagittal slices; slice thickness = 1 mm; FOV = 256 × 256 mm^2^; matrix size = 256 × 256; voxel size = 1 × 1 × 1 mm^3^; receiver bandwidth = 199 Hz/pixel) MPRAGE sequence was obtained to help fMRI data registration into MNI152 standard space.

### Functional MRI stimulation paradigm

Language lateralisation was assessed based on a verbal fluency task with a block design including seven cycles of 30-second-long rest alternating with 30-second-long silent word generation task according to given initial letters presented by MRI-compatible electrostatic headphones (NordicNeuroLab, Bergen, Norway). For a more detailed description of the paradigm see the study by Perlaki *et al*.^23^

### Functional MRI data processing and language laterality index calculation

The analysis of fMRI data was performed using FEAT v6.00 (FMRI Expert Analysis Tool), part of FSL v6.0.6.5 (FMRIB’s Software Library, www.fmrib.ox.ac.uk/fsl). Preprocessing steps included brain extraction (by BET), motion correction (by MCFLIRT), spatial smoothing with the default 5 mm full width at half maximum and high-pass temporal filtering (100 s cutoff). General linear model time-series statistical analyses were carried out using FMRIB’s Improved Linear Model with local autocorrelation correction.^24^ The first-level analysis included a single regressor of interest modelling the response to silent word generation and the temporal derivative of this waveform was also included in the design-matrix to correct for slight overall temporal shifts between the model and the data. To visually assess whether language areas were activated by the paradigm, first-level statistical maps were thresholded using clusters determined by *Z* > 5.0 and a corrected cluster significance threshold of *P* = 0.05.

The single-session data sets were registered into the MNI152 standard space using a two-stage process. First, the middle volume in the fMRI time-series of each subject was registered to that subject’s T1-weighted MPRAGE using BBR (6 degrees-of-freedom).^25^ Then, each subject’s MPRAGE image was registered to the 2 mm MNI152 standard-space T1-weighted template using a 12 degrees-of-freedom linear fit followed by non-linear registration (warp resolution = 10 mm).^26,27^ For each subject, the transformations of the two stages were combined into a single transformation and applied to individual first-level *t-map* to transform it straight into MNI152 space (trilinear interpolation used for resampling).

Language lateralisation assessment was focused on the Broca region, for which a symmetrical MNI152 standard space region-of-interest (ROI) mask was defined based on the Harvard-Oxford Cortical Structural Atlas provided by FSL. First, the bilateral Inferior Frontal Gyrus, pars triangularis and pars opercularis probabilistic masks of the atlas were thresholded at 10%, binarized, then incorporated into a single mask and finally recombined with its left-right flipped version. Based on this ROI for Broca and the MNI transformed individual *t-maps*, language laterality index (LI) was calculated for each subject using the LI-toolbox v1.3.2 with weighted mean and bootstrap methods.^28,29^

### Diffusion data processing and DTI-ALPS index calculations

**Figure 1:**
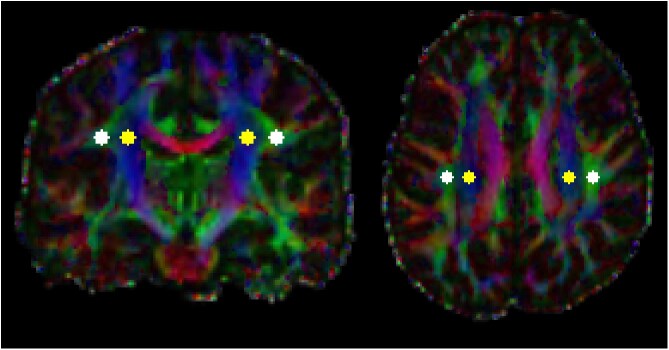
**Regions of interest positioning for DTI-ALPS index calculations.** Spherical ROIs in the association and projection tracts used for DTI-ALPS index calculations by Observer 2 are shown on top of the MNI-transformed coloured fractional anisotropy map in one of our subjects. Coronal and axial images represent slices at the centre of the ROIs.

After creating a binary brain mask for diffusion data by running FSL’s BET on the first b0 volume (i.e. *b*-value = 0 s/mm^2^),^30^ eddy current-induced distortions and subject movements were corrected as well as outlier slices with an average intensity at least 3 standard deviations lower than expected were detected and replaced with Gaussian Process prediction using the CUDA version of FSL’s eddy (eddy_cuda10.2).^31,32^ Diffusion tensor model was fitted on the preprocessed data using FSL’s dtifit command. The calculated FA map of each subject was linearly registered to the 1 mm JHU-ICBM-FA template supplied by FSL using 6 degrees-of-freedom.^26^ The resulting transformation matrix was applied to the diffusion tensor using the *vecreg* command line tool of FSL and diffusivity maps in the *x*-(left-right), *y*-(anterior-posterior) and *z*-(inferior-superior) directions (i.e. D_xx_, D_yy_, D_zz_) were extracted from the resampled tensor.^33^ For the calculation of DTI-ALPS indices, four spherical ROIs (each having a volume of 123 mm^3^) were placed in the projection and association fibres for both the left- and right-hemispheres at the level of upper part of lateral ventricle body. To minimize the bias from positioning the ROIs manually, the ROIs for ALPS calculations were placed independently by two independent Observers (Fig. 1). The DTI-ALPS index was calculated separately for the left- and right-hemispheres (i.e. left ALPS_index_ and right ALPS_index_) according to the following:


$$\begin{array}{rl} ALPS_{index} & =Mean(D_{xx,projection},D_{xx,association})/\\ & Mean(D_{yy,projection},D_{zz,association}), \end{array}$$

where D_xx,projection_ is the diffusivity along the *x*-direction averaged over the spherical ROI for projection fibres, D_xx,association_ is the *x*-axis diffusivity averaged over the ROI for association fibres, D_yy,projection_ is the *y*-axis diffusivity averaged over the ROI for projection fibres, while D_zz,association_ is the *z*-axis diffusivity averaged over the ROI for association fibres. A bilateral ALPS_index_ was also calculated by averaging the left- and right-sided indices. The bash codes for ALPS index calculations are provided within the Supplementary material.

### Statistical analysis

Statistical analyses were performed using IBM SPSS Statistics for Windows, Version 29.0 (IBM Corp., Armonk, NY, USA).

To assess the effects of handedness, multiple linear regression with left-, right-, or bilateral ALPS_index_ of each Observer as dependent variable and sex, age and EHI score as independent variables were performed. To assess the effects of LI, multiple linear regression with left-, right-, or bilateral ALPS_index_ of each Observer as dependent variable and sex, age and LI score as independent variables were performed. Since general linear models, with left-hemispheric ALPS_index_ of each Observer or the bilateral ALPS_index_ by Observer 1 as dependent variables indicated significant interaction effects between sex and LI (0.002 ≤ *P* ≤ 0.042), the multiple linear regression analyses were rather performed separately for males and females including age and LI as independent variables in the model. Significant outliers resulting in standardized residuals >±3 standard deviations from the multiple linear regression models were excluded (actual sample size always indicated). Wilcoxon signed ranked test was performed to assess ALPS_index_ differences between the hemispheres. Two-tailed *P*-values are reported and *P* ≤ 0.05 was the significance cutoff for all statistical tests.

Intraclass correlation coefficients (ICCs) were used to assess the consistency (i.e. systematic differences are irrelevant) and absolute agreement (i.e. systematic differences are relevant) between the DTI-ALPS indices by the two Observers. Two-way mixed model was selected, and both single- and average measures ICCs were obtained. As a rule of thumb, ICC values were classified as excellent (≥0.9), good (0.9 > ICC ≥ 0.8) and acceptable (0.8 > ICC ≥ 0.7).

## Results

Based on previously suggested optimal EHI score cut-off,^34^ our study group contained 45 right-handed (EHI > 60; 19 males; mean age: 27.96 ± 10.91, range: 18–64 years) and 37 atypical-handed (left- or mixed-handed; EHI ≤ 60; 16 males; mean age: 25.65 ± 9.18, range: 20–66 years) subjects. Based on the verbal fluency fMRI task, the LI ranged from −0.65 to +0.76, with a mean LI of 0.54 ± 0.31.

### Handedness

Multiple linear regression corrected for age and sex indicated that handedness was inversely related to the left ALPS_index_ by Observer 1 (*n* = 81, *P* = 0.030) and the bilateral ALPS_index_ by both Observers (*n* = 82, *P* = 0.025 and *P* = 0.044), suggesting that the more left-handed the subjects were, the higher the left and bilateral DTI-ALPS indices, and vice versa. As the significance pattern of the association between handedness and left ALPS_index_ was different for our two Observers, multiple linear regression analysis was also performed using the left ALPS_index_ averaged across the Observers as a dependent variable, indicating a trend-like inverse association with handedness (*n* = 80, *P* = 0.109). Yet, handedness was not significantly related to right-sided ALPS_index_ for any Observers (Table 1). Age and gender neither had significant effects from these multiple linear regression models for any of the DTI-ALPS indices/Observers (*P* ≥ 0.349). No significant interactions were found between any pairs of the independent variables (i.e. handedness, age, sex), except for the significant sex × age interaction term in case of left ALPS_index_ by Observer 2 (*P* = 0.027). The addition of this significant term to the model did not change the significance pattern (Table 1).

**Table 1:** Association between handedness and DTI-ALPS index

	Handedness	
	*P*-value	*t* (df)
Observer 1		
L_ALPS_index_	**0.030**	**−2.204** (**77)**
R_ALPS_index_	0.263	−1.128 (78)
B_ALPS_index_	**0**.**025**	**−2.293** (**78)**
Observer 2		
L_ALPS_index_	0.200 (0.201^a^)	−1.292 (76)
R_ALPS_index_	0.251	−1.155 (78)
B_ALPS_index_	**0**.**044**	**−2.048** (**78)**

*L_ALPS*_index_, *R_ALPS*_index_ and *B_ALPS*_index_ are left, right and bilateral DTI-ALPS indices, respectively.

*P*-value and *t* (df) are specific to handedness-related term in the multiple linear regression analyses adjusted for age and sex. *P*-values smaller than 0.05 are in bold.

^a^In this case *P*-value is reported when significant sex × age interaction term was also added to the model.

### Language lateralisation

Due to the significant interactions mentioned in the statistical analysis (i.e. sex × LI), LI-related statistics were rather performed separately for males and females. In females, multiple linear regression analysis corrected for age indicated that LI was inversely related to the left ALPS_index_ (*n* = 47, *P* < 0.001) and the bilateral ALPS_index_ (*n* = 47, *P* ≤ 0.003) by both observers, suggesting that the more right-lateralized (i.e. atypical) language processing was indicative of higher left and bilateral ALPS_index_, and vica versa. However, language lateralisation was not significantly associated with the right ALPS_index_. In males, LI was not related to any of the DTI-ALPS indices (Table 2). Age had no significant effects in these models for any of the sexes/DTI-ALPS indices/Observers (*P* ≥ 0.107). No significant interactions were found between LI × age for any of the sexes/DTI-ALPS indices/observers (*P* ≥ 0.383).

**Table 2:** Association of language lateralisation with DTI-ALPS index

	Males, lateralisation index		Females, lateralisation index	
	*P*-value	*t* (df)	*P*-value	*t* (df)
Observer 1				
L_ALPS_index_	0.527	0.639 (32)	**<0.001**	**−4.535** (**44)**
R_ALPS_index_	0.843	−0.200 (32)	0.211	−1.270 (44)
B_ALPS_index_	0.812	0.239 (32)	**0**.**001**	**−3.447** (**44)**
Observer 2				
L_ALPS_index_	0.613	0.511 (32)	**<0.001 (<0.001^a^)**	**−3.925** (**44)**
R_ALPS_index_	0.390	−0.872 (32)	0.200	−1.301 (44)
B_ALPS_index_	0.790	−0.269 (32)	**0**.**003**	**−3.094** (**44)**

*L_ALPS*_index_, *R_ALPS*_index_ and *B_ALPS*_index_ are left, right and bilateral DTI-ALPS indices, respectively.

*P*-value and *t* (df) are specific to language lateralisation-related term in the multiple linear regression analyses adjusted for age. *P*-values smaller than 0.05 are in bold.

^a^In this case *P*-value is reported after square-root transforming the ALPS_index_ in order to deal with slight non-normality of the standardized residuals (Shapiro-Wilk test, *P* = 0.048).

Since handedness may modulate LI to some extent (i.e. higher incidence of atypical language lateralisation in left-handers),^23^ as an exploratory analysis, we also examined whether the inclusion of handedness as an additional covariate affects our LI-related results in women. Although the significance pattern of LI-related results in women was not changed by adding handedness to the model, we found a significant interaction between handedness and LI for the left-hemispheric ALPS_index_ by Observer 1 (*P* = 0.021), suggesting that it is statistically more reliable to examine the effects of LI separately for right-handed and atypical-handed (i.e. mixed- and left-handed) female groups. Repeating multiple linear regression analyses separately for the right-handed (EHI >+60; *n* = 26) and atypical-handed (left- or mixed-handed; EHI ≤ 60; *n* = 21) female subgroups, LI remained negatively related to the left- and bilateral ALPS_index_ by both Observers in the atypical-handed female subgroup (Observer 1: *t* = −4.947, *P* < 0.001 and *t* = −3.867, *P* = 0.001 respectively; Observer 2: *t* = −3.927, *P* < 0.001 and *t* = −3.248, *P* = 0.004 respectively), but not in the right-handed subgroup (*P* ≥ 0.126). We did not find significant interactions between LI × age or significant effect of age from these models either (*P* ≥ 0.185).

### Interobserver agreement

The consistency and the absolute agreement among the two observers were good for all three versions of the ALPS_index_ (i.e. left, right and bilateral) when using the Single Measures ICCs and excellent when using the Average Measures ones (Table 3).

**Table 3:** Intraclass correlation coefficients between the two observers for DTI-ALPS indices

	L_ALPS_index_		R_ALPS_index_		B_ALPS_index_	
ICC	Absolute agreement	Consistency	Absolute agreement	Consistency	Absolute agreement	Consistency
Single Measures	0.857	0.856	0.843	0.845	0.898	0.899
Average Meaures	0.923	0.922	0.915	0.916	0.946	0.947

ICC intraclass correlation coefficient; *L_ALPS*_index_, *R_ALPS*_index_ and *B_ALPS*_index_ are the left, right and bilateral DTI-ALPS indices, respectively. Single- and average measures ICCs were obtained based on two-way mixed model.

### Interhemispheric differences in DTI-ALPS index

Left hemispheric ALPS_index_ was significantly higher than the right one for both observers (*n* = 82, *z* = −6.109, *P* < 0.001 and *z* = −6.474, *P* < 0.001) even if considering males (*n* = 35, *z* = −4.291, *P* < 0.001 and *z* = −4.390, *P* < 0.001) and females (*n* = 47, *z* = −4.318, *P* < 0.001 and *z* = −4.741, *P* < 0.001) separately. The leftward dominance of ALPS_index_ was also significant for both observers, when right-handed (EHI > 60; *n* = 45, *P* < 0.001), atypical-handed (left- or mixed-handed; EHI ≤ 60; *n* = 37; *P* < 0.001), mixed-handed (−60 ≤ EHI ≤ 60; *n* = 26; *P* < 0.001) or the pure left-handed (EHI ≤ −60; *n* = 11; *P* = 0.033 and *P* = 0.050 for Observer 1 and Observer 2) groups—defined based on previously suggested optimal EHI score cut-off^34^—were examined separately.

## Discussion

The present study aimed to analyze the relationship of the DTI-ALPS index with handedness and language lateralisation. Since there is no clear conclusion in the literature whether the index should be extracted only from the left-hemisphere, averaged over the two hemispheres or assessed separately for both hemispheres,^8,9,21,35^ all three versions of the index were examined.

Both handedness and language lateralisation were inversely related to the left- and/or bilateral ALPS indices, but not to the right-hemispheric one. Although the exact influence of handedness/LI on ALPS_index_ was not examined previously, some studies tried to address the potential confounding effects of handedness by including only right-handed subjects.^36^ Examining a right-handed population does not automatically guarantee that the LI-related changes of DTI measures disappear as demonstrated by Ocklenburg *et al*.^37^ who found correlation between LI and the strength of fractional anisotropy asymmetries of the arcuate fasciculus in a right-handed population. However, our results proved that the examination of right-handed subjects exclusively is indeed an effective way to eliminate the effects of LI on ALPS_index_ (i.e. in our right-handed female subgroup, LI was not related to the left- or bilateral ALPS_index_), which can also be intuitively expected given that, in general, 94–96% of right-handers show left-hemispheric language dominance.^23^

A considerable proportion of ALPS studies are retrospective, possibly without information on handedness/LI. Considering the lack of influence of handedness/LI on the right ALPS_index_, the right-hemispheric ALPS_index_ may be preferred in such situations. Moreover, investigating the right ALPS_index_, while also keeping left handers in the study, could be an effective way to increase the number of subjects without introducing serious confounding effects. However, it is important to note that this suggestion somewhat contrasts with the original DTI-ALPS study, suggesting that the index should be measured in the left hemisphere, which is probably why early ALPS studies focused less on the right side.^8^ In certain cases (including our present study) left- and right hemispheric indices may behave differently.^38^ Previous studies also reported discrepant results for the two hemispheres (e.g. only the left hemispheric ALPS_index_, not the right one, was reduced in patients with spontaneous intracerebral haemorrhage in the left hemisphere,^38^ patients with left temporal lobe epilepsy^17^ or early stage Parkinson’s disease patients^39^), suggesting that ALPS_index_ may change somewhat independently in the two hemispheres and thus should be investigated separately for the two hemispheres. We are not aware of any human or animal studies investigating the lateralisation of the glymphatic system using methods more convincing than DTI-ALPS. However, if future studies were to show the absence of glymphatic lateralisation, the independent changes of DTI-ALPS between the two hemispheres would provide additional support that DTI-ALPS index is unlikely to represent glymphatic activity.

While the validity of DTI-ALPS index as a marker of glymphatic brain clearance is questioned by some researchers,^10,40^ we cannot exclude the possibility that changes in glymphatic system function still underlie our correlations. However, it would be imprudent to interpret our correlations based on the very simplistic view of DTI-ALPS index as a marker of glymphatic function, i.e. atypical language dominance associated with increased glymphatic function in the left hemisphere.

Our results may be explained in another and potentially more plausible way, by structural differences related to language lateralisation, which could influence the ALPS_index_ value. Although the white matter correlates of LI have not been very thoroughly examined, the superior longitudinal and arcuate fasciculus (SLF and AF; association tracts) have been probably most frequently studied in this regard.^41,42^ Unfortunately, due to the close proximity and the potential spatial overlap of SLF and AF^43^ and because their names are often used interchangeably in the literature, previous studies may represent results mixed from both tracts.^23,41^ The normal left-right asymmetry of AF using both microstructural (i.e. FA = fractional anisotropy) and macrostructural (i.e. volume) white matter measures as well as the relevance of left AF in language functions have been demonstrated.^42^ Moreover, significant positive correlations between FA lateralisation in language-related pathways and fMRI activation asymmetries during language tasks have been reported,^37,42,44^ indicating that more pronounced left-greater-than-right FA asymmetry means stronger leftward fMRI lateralisation. Though not consistently observed in all previous studies, but the correlation between the laterality of AF volume and language lateralisation has been also demonstrated in both adults^45^ and preadolescent children.^46^ Based on this latter study, this correlation could be at least partially driven by the positive association reported between left AF volume and LI,^46^ that may potentially explain the direction of our inverse correlations between the left ALPS_index_ and LI. Although our fixed-size ROIs were carefully positioned in the centre of relevant tracts for ALPS calculations, it can happen that smaller left association tracts in subjects with smaller LI may result in partial volume effects from the adjacent subcortical fibres, whose main diffusion axis is in the *x*-direction. If the subcortical fibres are partially mixed, the ALPS_index_ will increase as suggested by Taoka *et al*.^13^ However, it should be noted that most of previous studies examined the AF as a whole (or divided it into a few segments), making it difficult to unequivocally conclude about LI-related diameter changes of AF in the short section where our ALPS ROIs were placed. Clearly, further prospective studies using high spatial and angular resolution advanced diffusion techniques combined with tractography methods (e.g. XTRACT, TRACULA) are needed, to specifically address the association between the final DTI-ALPS index and the morphological, micro- and macrostructural properties of AF locally measured at the level of ALPS ROIs.

DTI-ALPS index can also be influenced by many other factors, including the perivascular spaces (PVS) as shown by some studies detecting inverse relationship between enlarged PVS (EPVS) and the index.^39,47^ However, this correlation is not strong enough to present in all scenarios^48^ and its presence may also be dependent on the anatomical location of PVS measurements (i.e. ALPS_index_ negatively correlated with EPVS in the basal ganglia versus uncorrelated with EPVS in the centrum semiovale).^49^ Asymmetric distribution of EPVS has been described in temporal lobe, post-traumatic and post-stroke epilepsy,^17^ but we are not aware of studies about the lateralized distribution of PVS related to handedness or LI in healthy normal population. Although we cannot exclude the role of PVS in our results, it should be noted that typically only the PVS enlargement is detected on MRI scans and most PVS remain invisible,^13^ making it challenging to even plan future studies on the relationships among handedness/LI, PVS and ALPS_index_ in a healthy normal population.

Like Zhang *et al*.^38^ we can also speculate that different findings in the left- and right hemispheres might be associated with the separate blood supply of the two hemispheres and with the potential vascular correlates of handedness and cerebral dominance.^50,51^ However, further studies are needed to justify the background of these speculations.

Both ICCs for consistency and absolute agreement were good to excellent for all three versions of the ALPS_index_, indicating high inter-rater reliability of our measures. Unfortunately, previous articles providing ICCs for ALPS_index_ between observers rarely describe the exact type of the reported ICC values (e.g. Single- versus Average Measures, Absolute agreement versus consistency, 1-way random versus 2-way random effects versus 2-way mixed effects), making it rather difficult to compare our ICCs with those in the literature.^33,38,52,53^ However, despite our high inter-observer reliability, instead of averaging the DTI-ALPS measures between the observers, we ran all statistics separately for the two observers to be even more confident that the results are robust and observer independent. In this sense, it is important to mention that the inverse association between handedness and the left ALPS_index_ was the only one of our results that showed some observer dependence (i.e. significant for Observer 1 versus non-significant for Observer 2), and working with a left ALPS_index_ averaged across observers resulted in only a non-significant trend for this inverse association.

Left hemispheric ALPS_index_ was significantly higher than the right one. The leftward asymmetry of ALPS_index_ in normal adult brain has also been described by others and suggested that it may be associated with the structural and functional asymmetries of the brain.^17,36^ Wang *et al*.^36^ suggested that one possible reason for the leftward dominance of ALPS_index_ may come from right-handedness of their subjects, but here we proved that leftward dominance was present in the non-right handed subgroups as well.

In our sample, age had no significant effect on the examined DTI-ALPS indices. While the inverse association between age and ALPS_index_ was described by previous reports,^54^ Taoka *et al*.^20^ demonstrated that the age-related association is rather non-monotonic. Based on their statistical analysis over age groups created on decades, no significant differences were observed among the 10’s, 20’s, 30’s, 50’s and 60’s generations, that represent 94% of our subjects, and the second-degree association found is mainly driven by the 40’s and 70’s generation. However, in our sample only 6% of subjects fell into the 40’s generation and nobody into the 70’s one. In addition, <10% of our sample was older than 40 years, not allowing us to examine how the index changes in older generations.

Sex differences in ALPS_index_ is rather controversial in the literature. Similar to our present study, several previous studies found a non-significant difference between males and females,^54^ but contradictory results suggesting larger ALPS_index_ in females were also reported, including a study with very large sample size based on UK biobank.^55^ However, according to a recent study, head-size is a major contributing factor to sex difference in ALPS_index_, and when the statistics are corrected for intracranial volume, males and females are no longer statistically different.^56^ Based on this, it can be also speculated that without a head-size correction, the presence or absence of difference depends on the similarity in head size between the involved male and female groups. This phenomenon is not unique to the ALPS_index_, but the hippocampal morphology shows the same behaviour (i.e. sex difference in the hippocampal volume depends on whether head-size correction is applied or not; and no difference is observed between head-size matched males and females even when no head-size correction is applied).^57^

In our cohort, a significant *sex* × *language lateralisation* interaction was found. This interaction suggests different association between LI and ALPS_index_ for males and females, that was later also confirmed by results of the separate statistical analyses in men and women (i.e. no relationship between LI and ALPS_index_ for males and significant inverse association for females). This specific interaction was not examined/reported previously, thus further studies needed for better understanding the exact mechanisms underlying it.

### Methodological considerations

In the present study we retrospectively evaluated DTI measurements acquired with *b* = 700 s/mm^2^, which is a little <*b* = 1000 s/mm^2^ used in many ALPS studies. However, it is not verified whether this latter is the optimal b-value for ALPS_index_.^13^ The ROIs for ALPS calculations were placed manually, that may add subjectivity to our measurements. Nevertheless, we repeated all statistical analyses separately for our two observers, and our findings were rather robust to be observer-independent. The theoretical basis of DTI-ALPS index as a marker of glymphatic system is criticized by recent studies.^10,40^ Given the complexity of the glymphatic system, like Haller *et al*.^10^ we also have doubts about the ability of this simple measure to fairly represent the whole glymphatic system. However, the changes of ALPS_index_ in various diseased conditions and its correlation with cognitive functions make it a valuable measure and any new studies about its correlates may help to find out what it truly represents and to identify its potential confounders.

In this study, we manually placed the ROIs at the projection and association fibres. The applied method (i.e. using vecreg function of FSL to register tensor data into standard space combined with manual ROI placement) demonstrated good-to-excellent inter-rater reliability on our data, and Tatekawa *et al*.^33^ also reported both good-to-excellent intra- and inter-reliabilities for this method. However, future multi-site ALPS studies targeting large sample sizes may require validated automated methods in order to reduce human resources (e.g. https://github.com/gbarisano/alps).^21,58^

Since <10% of our sample were older than 40 years, and because we also corrected for age in the statistics, age-related confounding effects are unlikely to affect our results. On the other hand, it may be possible that our results are not directly generalisable for the whole (including the older) population.

Since different language fMRI tasks may lead to activation of different language regions (e.g. Broca’s versus Wernicke’s area) that may be lateralized in varying degrees,^59^ future studies are warranted to test the generalizability of our findings using other types of language fMRI tasks.

In the present study, we also reported results for the bilateral ALPS_index_ in addition to left- and right hemispheric versions of the index. However, associations between handedness/LI and the bilateral ALPS_index_ are probably driven by the left hemisphere making the inclusion of bilateral results somewhat questionable. While we suggest that keeping the left and right hemispheric indices separate without averaging them would enhance clarity, the bilateral index was suggested to be more reproducible,^60^ which makes it rather difficult to simply ignore it in the present or future studies.

Although all of our analyses were controlled for age and gender, which were shown to be the two strongest independent predictors of ALPS index among a variety of demographic and vascular factors,^19^ future prospective studies should definitely consider a broader range of confounding factors, especially the vascular risk factors. Nevertheless, given the absence of any studies linking handedness/LI to vascular risk factors, it is unlikely that our findings for handedness/LI are primarily driven by these factors.

## Conclusions

In conclusion, both language laterality and handedness can affect DTI-ALPS index that should be considered in future studies. To control this confounding factor, handedness/LI-related changes should be statistically modelled. If this is not possible, DTI-ALPS index should either be derived from the right hemisphere, or the subject population should contain only right-handed individuals.

## Supplementary Material

fcaf252_Supplementary_Data

## Data Availability

Data supporting the findings of this study are available upon reasonable request to the corresponding author.

## References

[1] Xu Y, Cheng L, Yuan L, Yi Q, Xiao L, Chen H. Progress on brain and ocular lymphatic system. Biomed Res Int. 2022;2022:6413553. 36425338 10.1155/2022/6413553 PMC9681545

[2] Iliff JJ, Wang M, Liao Y, et al A paravascular pathway facilitates CSF flow through the brain parenchyma and the clearance of interstitial solutes, including amyloid β. Sci Transl Med. 2012;4(147):147ra111. 10.1126/scitranslmed.3003748 PMC3551275 22896675

[3] Louveau A, Smirnov I, Keyes TJ, et al Structural and functional features of central nervous system lymphatic vessels. Nature. 2015;523(7560):337–341. 26030524 10.1038/nature14432 PMC4506234

[4] Iliff JJ, Lee H, Yu M, et al Brain-wide pathway for waste clearance captured by contrast-enhanced MRI. J Clin Invest. 2013;123(3):1299–1309. 23434588 10.1172/JCI67677 PMC3582150

[5] Ringstad G, Valnes LM, Dale AM, et al Brain-wide glymphatic enhancement and clearance in humans assessed with MRI. JCI Insight. 2018;3(13):e121537. 29997300 10.1172/jci.insight.121537 PMC6124518

[6] Kamagata K, Saito Y, Andica C, et al Noninvasive magnetic resonance imaging measures of glymphatic system activity. J Magn Reson Imaging. 2024;59(5):1476–1493. 37655849 10.1002/jmri.28977

[7] Taoka T, Naganawa S. Glymphatic imaging using MRI. J Magn Reson Imaging. 2020;51(1):11–24. 31423710 10.1002/jmri.26892

[8] Taoka T, Masutani Y, Kawai H, et al Evaluation of glymphatic system activity with the diffusion MR technique: Diffusion tensor image analysis along the perivascular space (DTI-ALPS) in Alzheimer’s disease cases. Jpn J Radiol. 2017;35(4):172–178. 28197821 10.1007/s11604-017-0617-z

[9] Zhang W, Zhou Y, Wang J, et al Glymphatic clearance function in patients with cerebral small vessel disease. Neuroimage. 2021;238:118257. 34118396 10.1016/j.neuroimage.2021.118257

[10] Haller S, Moy L, Anzai Y. Evaluation of diffusion tensor imaging analysis along the perivascular space as a marker of the glymphatic system. Radiology. 2024;310(1):e232899. 38289215 10.1148/radiol.232899

[11] Wright AM, Wu Y-C, Chen N-K, Wen Q. Exploring radial asymmetry in MR diffusion tensor imaging and its impact on the interpretation of glymphatic mechanisms. J Magn Reson Imaging. 2024;60(4):1432–1441. 38156600 10.1002/jmri.29203 PMC11213825

[12] Georgiopoulos C, Werlin A, Lasic S, et al Diffusion tensor imaging along the perivascular space: The bias from crossing fibres. Brain Commun. 2024;6(6):fcae421. 39713238 10.1093/braincomms/fcae421 PMC11660947

[13] Taoka T, Ito R, Nakamichi R, Nakane T, Kawai H, Naganawa S. Diffusion tensor image analysis ALong the perivascular space (DTI-ALPS): Revisiting the meaning and significance of the method. Magn Reson Med Sci. 2024;23(3):268–290. 38569866 10.2463/mrms.rev.2023-0175 PMC11234944

[14] Carotenuto A, Cacciaguerra L, Pagani E, Preziosa P, Filippi M, Rocca MA. Glymphatic system impairment in multiple sclerosis: Relation with brain damage and disability. Brain. 2022;145(8):2785–2795. 34919648 10.1093/brain/awab454

[15] Yang D-X, Sun Z, Yu M-M, et al Associations of MRI-derived glymphatic system impairment with global white matter damage and cognitive impairment in mild traumatic brain injury: A DTI-ALPS study. J Magn Reson Imaging. 2024;59(2):639–647. 37276070 10.1002/jmri.28797

[16] Lee DA, Park BS, Ko J, et al Glymphatic system dysfunction in temporal lobe epilepsy patients with hippocampal sclerosis. Epilepsia Open. 2022;7(2):306–314. 35305294 10.1002/epi4.12594 PMC9159256

[17] Zhao X, Zhou Y, Li Y, et al The asymmetry of glymphatic system dysfunction in patients with temporal lobe epilepsy: A DTI-ALPS study. J Neuroradiol. 2023;50(6):562–567. 37301366 10.1016/j.neurad.2023.05.009

[18] Bae YJ, Kim JM, Choi BS, et al Glymphatic function assessment in Parkinson’s disease using diffusion tensor image analysis along the perivascular space. Park Relat Disord. 2023;114:105767. 10.1016/j.parkreldis.2023.105767 37523953

[19] Zhang Y, Zhang R, Ye Y, et al The influence of demographics and vascular risk factors on glymphatic function measured by diffusion along perivascular space. Front Aging Neurosci. 2021;13:693787. 34349635 10.3389/fnagi.2021.693787 PMC8328397

[20] Taoka T, Ito R, Nakamichi R, et al Diffusion-weighted image analysis along the perivascular space (DWI–ALPS) for evaluating interstitial fluid status: Age dependence in normal subjects. Jpn J Radiol. 2022;40(9):894–902. 35474438 10.1007/s11604-022-01275-0 PMC9441421

[21] Liu X, Barisano G, Shao X, et al Cross-vendor test-retest validation of diffusion tensor image analysis along the perivascular space (DTI-ALPS) for evaluating glymphatic system function. Aging Dis. 2024;15(4):1885–1898. 37307817 10.14336/AD.2023.0321-2 PMC11272201

[22] Oldfield RC . The assessment and analysis of handedness: The Edinburgh inventory. Neuropsychologia. 1971;9(1):97–113. 5146491 10.1016/0028-3932(71)90067-4

[23] Perlaki G, Horvath R, Orsi G, et al White-matter microstructure and language lateralization in left-handers: A whole-brain MRI analysis. Brain Cogn. 2013;82(3):319–328. 23792788 10.1016/j.bandc.2013.05.005

[24] Woolrich MW, Ripley BD, Brady M, Smith SM. Temporal autocorrelation in univariate linear modeling of FMRI data. Neuroimage. 2001;14(6):1370–1386. 11707093 10.1006/nimg.2001.0931

[25] Greve DN, Fischl B. Accurate and robust brain image alignment using boundary-based registration. Neuroimage. 2009;48(1):63–72. 19573611 10.1016/j.neuroimage.2009.06.060 PMC2733527

[26] Jenkinson M, Bannister P, Brady M, Smith S. Improved optimization for the robust and accurate linear registration and motion correction of brain images. Neuroimage. 2002;17(2):825–841. 12377157 10.1016/s1053-8119(02)91132-8

[27] Andersson JLR, Jenkinson M, Smith S. Non-linear registration aka spatial normalisation FMRIB Technial report TR07JA2. FMRIB Anal Gr Univ Oxford, 2007.

[28] Wilke M, Lidzba K. LI-tool: A new toolbox to assess lateralization in functional MR-data. J Neurosci Methods. 2007;163(1):128–136. 17386945 10.1016/j.jneumeth.2007.01.026

[29] Wilke M, Schmithorst VJ. A combined bootstrap/histogram analysis approach for computing a lateralization index from neuroimaging data. Neuroimage. 2006;33(2):522–530. 16938470 10.1016/j.neuroimage.2006.07.010

[30] Smith SM . Fast robust automated brain extraction. Hum Brain Mapp. 2002;17(3):143–155. 12391568 10.1002/hbm.10062 PMC6871816

[31] Andersson JLR, Sotiropoulos SN. An integrated approach to correction for off-resonance effects and subject movement in diffusion MR imaging. Neuroimage. 2016;125:1063–1078. 26481672 10.1016/j.neuroimage.2015.10.019 PMC4692656

[32] Andersson JLR, Graham MS, Zsoldos E, Sotiropoulos SN. Incorporating outlier detection and replacement into a non-parametric framework for movement and distortion correction of diffusion MR images. Neuroimage. 2016;141:556–572. 27393418 10.1016/j.neuroimage.2016.06.058

[33] Tatekawa H, Matsushita S, Ueda D, et al Improved reproducibility of diffusion tensor image analysis along the perivascular space (DTI-ALPS) index: An analysis of reorientation technique of the OASIS-3 dataset. Jpn J Radiol. 2023;41(4):393–400. 36472803 10.1007/s11604-022-01370-2 PMC10066136

[34] Dragovic M . Categorization and validation of handedness using latent class analysis. Acta Neuropsychiatr. 2004;16(4):212–218. 26984309 10.1111/j.0924-2708.2004.00087.x

[35] Morita Y, Kamagata K, Andica C, et al Glymphatic system impairment in nonathlete older male adults who played contact sports in their youth associated with cognitive decline: A diffusion tensor image analysis along the perivascular space study. Front Neurol. 2023;14:1100736. 36873446 10.3389/fneur.2023.1100736 PMC9977161

[36] Wang L, Qin Y, Li X, et al Glymphatic-system function is associated with addiction and relapse in heroin dependents undergoing methadone maintenance treatment. Brain Sci. 2023;13(9):1292. 37759893 10.3390/brainsci13091292 PMC10526898

[37] Ocklenburg S, Hugdahl K, Westerhausen R. Structural white matter asymmetries in relation to functional asymmetries during speech perception and production. Neuroimage. 2013;83:1088–1097. 23921095 10.1016/j.neuroimage.2013.07.076

[38] Zhang C, Sha J, Cai L, et al Evaluation of the glymphatic system using the DTI-ALPS index in patients with spontaneous intracerebral haemorrhage. Oxid Med Cell Longev. 2022;2022:2694316. 35847591 10.1155/2022/2694316 PMC9277160

[39] Shen T, Yue Y, Ba F, et al Diffusion along perivascular spaces as marker for impairment of glymphatic system in Parkinson’s disease. NPJ Park Dis. 2022;8(1):174. 10.1038/s41531-022-00437-1 PMC9772196 36543809

[40] Ringstad G . Glymphatic imaging: A critical look at the DTI-ALPS index. Neuroradiology. 2024;66(2):157–160. 38197950 10.1007/s00234-023-03270-2

[41] Budisavljevic S, Castiello U, Begliomini C. Handedness and white matter networks. Neuroscientist. 2021;27(1):88–103. 32723129 10.1177/1073858420937657

[42] Ocklenburg S, Friedrich P, Güntürkün O, Genç E. Intrahemispheric white matter asymmetries: The missing link between brain structure and functional lateralization? Rev Neurosci. 2016;27(5):465–480. 26812865 10.1515/revneuro-2015-0052

[43] Janelle F, Iorio-Morin C, D’amour S, Fortin D. Superior longitudinal fasciculus: A review of the anatomical descriptions with functional correlates. Front Neurol. 2022;13:794618. 35572948 10.3389/fneur.2022.794618 PMC9093186

[44] Powell HWR, Parker GJM, Alexander DC, et al Hemispheric asymmetries in language-related pathways: A combined functional MRI and tractography study. Neuroimage. 2006;32(1):388–399. 16632380 10.1016/j.neuroimage.2006.03.011

[45] Silva G, Citterio A. Hemispheric asymmetries in dorsal language pathway white-matter tracts: A magnetic resonance imaging tractography and functional magnetic resonance imaging study. Neuroradiol J. 2017;30(5):470–476. 28699372 10.1177/1971400917720829 PMC5602342

[46] Sreedharan RM, Menon AC, James JS, Kesavadas C, Thomas SV. Arcuate fasciculus laterality by diffusion tensor imaging correlates with language laterality by functional MRI in preadolescent children. Neuroradiology. 2015;57(3):291–297. 25467219 10.1007/s00234-014-1469-1

[47] Ma X, Li S, Li C, et al Diffusion tensor imaging along the perivascular space Index in different stages of Parkinson’s disease. Front Aging Neurosci. 2021;13:773951. 34867300 10.3389/fnagi.2021.773951 PMC8634754

[48] Butler T, Zhou L, Ozsahin I, et al Glymphatic clearance estimated using diffusion tensor imaging along perivascular spaces is reduced after traumatic brain injury and correlates with plasma neurofilament light, a biomarker of injury severity. Brain Commun. 2023;5(3):fcad134. 37188222 10.1093/braincomms/fcad134 PMC10176239

[49] Liu H, Yang S, He W, et al Associations among diffusion tensor image along the perivascular space (DTI-ALPS), enlarged perivascular space (ePVS), and cognitive functions in asymptomatic patients with carotid plaque. Front Neurol. 2022;12:789918. 35082748 10.3389/fneur.2021.789918 PMC8785797

[50] Jansen van Vuuren A, Saling MM, Ameen O, Naidoo N, Solms M. Hand preference is selectively related to common and internal carotid arterial asymmetry. Laterality. 2017;22(4):377–398. 27380444 10.1080/1357650X.2016.1205596

[51] Jansen van Vuuren A, Saling M, Rogerson S, Anderson P, Cheong J, Solms M. Vascular underpinnings of cerebral lateralisation in the neonate. Symmetry (Basel). 2024;16(2):161.

[52] Ruan X, Huang X, Li Y, Li E, Li M, Wei X. Diffusion tensor imaging analysis along the perivascular space index in primary Parkinson’s disease patients with and without freezing of gait. Neuroscience. 2022;506:51–57. 36341724 10.1016/j.neuroscience.2022.10.013

[53] Pang H, Wang J, Yu Z, et al Glymphatic function from diffusion-tensor MRI to predict conversion from mild cognitive impairment to dementia in Parkinson’s disease. J Neurol. 2024;271(8):5598–5609. 38913186 10.1007/s00415-024-12525-8 PMC11319419

[54] Villacis G, Schmidt A, Rudolf JC, et al Evaluating the glymphatic system via magnetic resonance diffusion tensor imaging along the perivascular spaces in brain tumor patients. Jpn J Radiol. 2024;42:1146–1156. 38819694 10.1007/s11604-024-01602-7 PMC11442616

[55] Clark O, Delgado-Sanchez A, Cullell N, Correa SAL, Krupinski J, Ray N. Diffusion tensor imaging analysis along the perivascular space in the UK biobank. Sleep Med. 2024;119:399–405. 38772221 10.1016/j.sleep.2024.05.007

[56] Ozsahin I, Zhou L, Wang X, et al Diffusion tensor imaging along perivascular spaces (DTI-ALPS) to assess effects of age, sex, and head size on interstitial fluid dynamics in healthy subjects. J Alzheimers Dis Rep. 2024;8(1):355–361. 38405348 10.3233/ADR-230143 PMC10894616

[57] Perlaki G, Orsi G, Plozer E, et al Are there any gender differences in the hippocampus volume after head-size correction? A volumetric and voxel-based morphometric study. Neurosci Lett. 2014;570:119–123. 24746928 10.1016/j.neulet.2014.04.013

[58] Saito Y, Kamagata K, Andica C, et al Reproducibility of automated calculation technique for diffusion tensor image analysis along the perivascular space. Jpn J Radiol. 2023;41(9):947–954. 37162692 10.1007/s11604-023-01415-0

[59] Desai VR, Vedantam A, Lam SK, et al Language lateralization with resting-state and task-based functional MRI in pediatric epilepsy. J Neurosurg Pediatr. 2019;23(2):171–177. 30485177 10.3171/2018.7.PEDS18162

[60] Taoka T, Ito R, Nakamichi R, et al Reproducibility of diffusion tensor image analysis along the perivascular space (DTI-ALPS) for evaluating interstitial fluid diffusivity and glymphatic function: CHanges in Alps index on Multiple conditiON acquIsition eXperiment (CHAMONIX) study. Jpn J Radiol. 2022;40(2):147–158. 34390452 10.1007/s11604-021-01187-5 PMC8803717

